# Molecular Characteristics of the Equine Periodontal Ligament

**DOI:** 10.3389/fvets.2017.00235

**Published:** 2018-01-11

**Authors:** Antje Pöschke, Bastian Krähling, Klaus Failing, Carsten Staszyk

**Affiliations:** ^1^Institute of Veterinary-Anatomy, -Histology and -Embryology, Justus Liebig University Giessen, Giessen, Germany; ^2^Department of Biomathematics, Justus Liebig University Giessen, Giessen, Germany

**Keywords:** periodontal ligament, tendon, collagen, scleraxis, equine

## Abstract

The equine periodontal ligament (PDL) is a fibrous connective tissue that covers the intra-alveolar parts of the tooth and anchors it to the alveolar bone—it, therefore, provides a similar function to a tendinous structure. While several studies have considered the formation and structure of tendons, there is insufficient information particularly on the molecular composition of the PDL. Especially for the equine PDL, there is limited knowledge concerning the expression of genes commonly regarded as typical for tendon tissue. In this study, the gene expression of, e.g., *collagen type 1 alpha 1* (*COL1), collagen type 3 alpha 1 (COL3), scleraxis (SCX)*, and fibrocartilage markers was examined in the functional mature equine PDL compared with immature and mature equine tendon tissue. PDL samples were obtained from incisor, premolar, and molar teeth from seven adult horses. Additionally, tendon samples were collected from four adult horses and five foals at different sampling locations. Analyses of gene expression were performed using real-time quantitative polymerase chain reaction (qRT-PCR). Significantly higher expression levels of *COL1* and *3* were found in the mature equine PDL in comparison with mature tendon, indicating higher rates of collagen production and turnover in the mature equine PDL. The expression levels of *SCX*, a specific marker for tenogenic-differentiated cells, were on a similar level in functional mature PDL and in mature tendon tissue. Evidence of chondrogenic metaplasia, often found in tendon entheses or in pressurized regions of tendons, was not found in the mature equine PDL. The obtained results justify further experiments focused on the possible use of equine PDL cells for cell-based regenerative therapies.

## Introduction

The equine periodontal ligament (PDL) is part of a complex formation called the periodontium. The basic periodontium structure consists of four components termed the PDL, the alveolar bone, the dental cementum, and the gingiva (1, 2). Its central function is to fix the tooth in the alveolus. Moreover, it has the task of absorbing and discharging masticatory tension and compression forces (3–6). The PDL of brachydont (short-crowned) as well of that of hypsodont (high-crowned) species comprises collagen fibers surrounded by ground substance containing various types of cells, blood vessels, and nerves (7, 8). Equine hypsodont teeth are an adaption to the very abrasive silicate-rich food of herbivores (6). One major challenge for the equine PDL is to accomplish continuous eruption throughout the life of the animal, in response to a permanent occlusal wear of 3–4 mm per year (1, 9, 10). Thus, in horses, the components of the periodontium must enable tooth support while managing continuous tooth eruption. Consequently, a very dynamic system is needed, which is capable of both continuous collagen remodeling and permanent provision of a sufficient apparatus to withstand biomechanical loads similar to those in a load-bearing tendon.

Several studies in different species have addressed the collagen fiber apparatus and extracellular matrix (ECM) composition of tendons (11–14) and their entheses (15, 16). The distinctive formation of a tendinous structure is composed of numerous fiber bundles arranged along the long axis of the tendon embedded in a well-coordinated ECM (12, 17). COL1 is the primary protein in tendinous structures, accompanied to a much lesser extent by COL3 (13, 17, 18). The transcription factor scleraxis (SCX) plays a key role during tenogenic and ligamentogenic differentiation (19–23). As SCX is also expressed in mature tendinous tissue, it is generally accepted as a specific marker to identify differentiated tenogenic and ligamentogenic cells (22, 24).

Tendon and PDL tissues are subjected to tensile and shear forces, as well as compression loads in distinct regions, e.g., near entheses or where they wrap around bony or fibrous pulleys (15, 16, 25). Entheses are specialized fibrocartilaginous or fibrous regions where tendons and ligaments are attached to bone (15, 16). Local compression loads can trigger tendon tissue to produce cartilage-like matrix, which subsequently transforms into fibrocartilage tissue (15, 26). A predominant fibrocartilaginous area in the superficial digital flexor tendon is the place where it is overlying the metacarpophalangeal joint (15, 25, 27). In these regions, characteristic protein expression of type II collagen (COL2), cartilage oligomeric matrix protein (COMP), and aggrecan (ACAN) is present. Osteopontin (OPN), a protein related to osteogenesis, can also be found in the region of entheses (28).

During tendon maturation, cells that are capable of tenogenesis drift more and more into the background and shift into a status of limited metabolic activity (bradytrophy) (14, 29, 30). It is supposed that mature tendon tissue is a primarily static tissue with a low turnover and limited regenerative and reparative capacities (14, 18, 19, 31–34). Therefore, healing processes (for example after tendon injuries) represent a common orthopedic problem (19). The PDL has completely different characteristics—cellular components exist in large numbers with up to 50% of total volume (7, 35, 36), while extracellular components are subjected to constant synthesis and degradation, which is described as continuous remodeling (37, 38). In brachydont rodent molars, proliferation rates of about 2% have been observed (36, 37, 39, 40). Studies in the PDL of brachydont species, including humans, have revealed that cell turnover is balanced between proliferation and apoptosis (36). To maintain tissue homeostasis under physiological conditions, synthesis and degradation of periodontal structures must be harmonized (41, 42). Nevertheless, external forces, e.g., as a result of orthodontic tooth movement, increase the amount of periodontal remodeling in order to adapt the periodontal environment to the shifting teeth. It is supposed that the PDL of hypsodont equine teeth resembles the PDL of brachydont teeth under conditions of orthodontic tooth movement (38). It can, therefore, be assumed that in the equine PDL, processes run continuously, which only temporarily occur in tendons during tenogenesis or repair.

Thus, the aim of the present study was to compare the mature equine PDL with immature and mature equine tendon tissue with regard to the expression patterns of genes involved in tenogenesis. Based on these criteria, the question arises as to whether PDL-derived cells exhibit characteristics that can be generally useful in cell-based therapies for tendon diseases.

## Materials and Methods

### Animals and Sample Collection

Specimens of different tissues were taken from five foals and seven adult horses, which had died or were euthanized on humane grounds unrelated to this study at the Clinic of Maternity, Gynecology, and Andrology and at the Clinic for Horses—Internal Medicine, Faculty of Veterinary Medicine, Justus Liebig University Giessen, Germany. The animals were of different genders and ages (the age range of the foals was about 4 weeks pre-parturition to 2 days post-parturition, and the age range of the adult horses was from 7 to 27 years). Samples were taken immediately post euthanasia or post mortem within a time frame of 2 h. PDL samples were taken from incisor, premolar, and molar teeth of the left mandible (301, 306, 309) from seven adult horses. For sample collection, a 4.5 cm × 4.5 cm square plate was sawn out of the alveolar bone to get access to the PDL (oscillating saw HB 8891, HEBUmedial GmbH, Tutlingen, Germany). PDL tissue was collected by gently scraping of the tooth surface with scalpel and tweezer, avoiding to sample parts of the alveolar bone. PDL samples were obtained from the labial and the buccal side, respectively. Tendon samples [i.e., superficial and deep digital flexor tendon, common digital extensor tendon and accessory (check) ligament of the deep digital flexor tendon of the left forelimb and tendon of the sternomandibularis muscle] were taken from four of the seven adult horses (aged 18–27 years) and five foals (aged 4 weeks pre-parturition to 2 days post-parturition). Each tendon sample, e.g., superficial and deep digital flexor tendon as well as the common digital extensor tendon were sampled in the mid-metacarpus region, the accessory (check) ligament of the deep digital flexor tendon was sampled in the middle of its proximodistal extension and the tendon of the sternomandibularis muscle was sampled in the middle of its craniocaudal extension. Samples were taken from the tendon core.

### RNA Extraction

Periodontal ligament and tendon samples were transferred to 1.7 ml reaction tubes containing 1 ml peqGOLD^®^ TriFast™ (Peqlab, Biotechnology GmbH, Erlangen, Germany). Prior to RNA extraction, specimens were homogenized with an Ultra Turrax^®^ homogenizer. Total RNA was isolated from the cells according to the manufacturer’s protocol. The RNA concentration was measured using an absorption photometer (BioPhotometer, Eppendorf AG, Hamburg, Germany). To guarantee the purity of the RNA, the ratio of sample absorbance at 260 and 280 nm was calculated. RNA samples were immediately frozen in liquid nitrogen and then stored at −196°C.

### DNAse Treatment and Transcription

Isolated total RNA was used at a concentration of 200 ng/µl. Subsequently, genomic DNA was digested using DNAse I (Roche, Grenzach, Germany). Reverse transcription was performed using a reaction mix containing 50 U Reverse Transcriptase, RNAse Inhibitor (20 U/μl), Random Hexamers, dNTP mix, MgCl_2_, and PCR Buffer Gold (all products of Invitrogen—Life Technologies, Darmstadt, Germany). Negative control samples were employed, omitting reverse transcriptase from the reaction mixtures (minus-RT). Successful cDNA synthesis and absence of genomic DNA in minus-RT controls was tested using a qualitative PCR for glyceraldehyde-3-phoshpate dehydrogenase (GAPDH, forward: 5′-gcg tga acc acg aga aat atg a-3′ and reverse 5′-ggt ggt gca gga ggc att g-3′, Eurofins Genomics, Eberberg, Germany).

### Real-time Quantitative Polymerase Chain Reaction (qRT-PCR)

According to the manufacturer’s protocol, the qRT-PCR was performed with 1 µl cDNA and 12.5 µl RT^2^ SYBR Green Mastermix (Qiagen, Hilden, Germany) combined with 1 µl RT^2^ qPCR Primer Assay (Qiagen, Hilden, Germany) and 10.5 µl water, using a BioRad CFX96 Touch Real-time PCR Detection System (Bio-Rad Laboratories, Munich, Germany). An initial denaturation at 95.0°C for 10:00 min was followed by 40 cycles with two steps: 95.0°C for 0:15 min and 55.0°C for 0:30 min. The run was finished with a single step of 72.0°C for 0:30 min before a melt curve from 60.0°C to 95.0°C (increment 0.5°C), to ensure specificity, was carried out.

The following equine RT^2^ qPCR primer assays were used for analysis: *ACAN* (Qiagen-ID: PPE00103A), *COL1A1* (Qiagen-ID: PPE00104A), *COL2A1* (Qiagen-ID: PPE00009A), *COL3A1* (Qiagen-ID: PPE00310A), *COMP* (Qiagen-ID: PPE00133A), *OPN* (Qiagen-ID: PPE08493A), and *SCX* (Qiagen-ID: PPE06980A). As reference genes, *GAPDH* (Qiagen-ID: PPE00120A) and β*-Actin* (Qiagen-ID: PPE00105A) were selected.

For all genes, the same cDNA per probe was used to investigate the expression and all genes were tested in triplicate for each probe to avoid pipetting mistakes. A no-template-control was included for each gene to exclude contamination. Data were analyzed using the BioRad CFX Manager™ Software (version 3.0) applying the ΔΔCt-method for gene expression, relative to the two reference genes GAPDH and β-Actin. For inter-run calibration, cDNA from equine mesenchymal stroma cells was pooled and used as a PCR template in every run.

### Statistical Analysis

Statistical analyses were performed using the statistical program package BMDP (43). To identify differences in gene expression between the three differently located PDL samples (301, 306, 309) and, among the other, the five tendon sample locations in adult horses, we used one-way analysis of variance (ANOVA) with repeated measurements (program BMDP5V). Subsequently, the mean gene expression values in PDL and tendon samples from all locations were calculated for each horse. To detect differences in gene expression between mature PDL and mature tendon samples, a *t*-test for dependent samples was employed (program BMDP3D). A two-way ANOVA with repeated measurements with respect to location (program BMDP5V) was used to test for gene expression differences between mature and immature tendon samples, also taking differences between the locations into account. Differences between mature PDL and immature tendon were assessed descriptively (without statistical analysis) because of not comparable conditions (different tissues and ages). The outcome of the statistical tests was considered to be significant when *p* ≤ 0.05. Boxplot generation was performed using GraphPad Prism 6 (GraphPad Software, La Jolla, CA, USA).

## Results

### One-Way ANOVA with Repeated Measures

**Figure 1:**
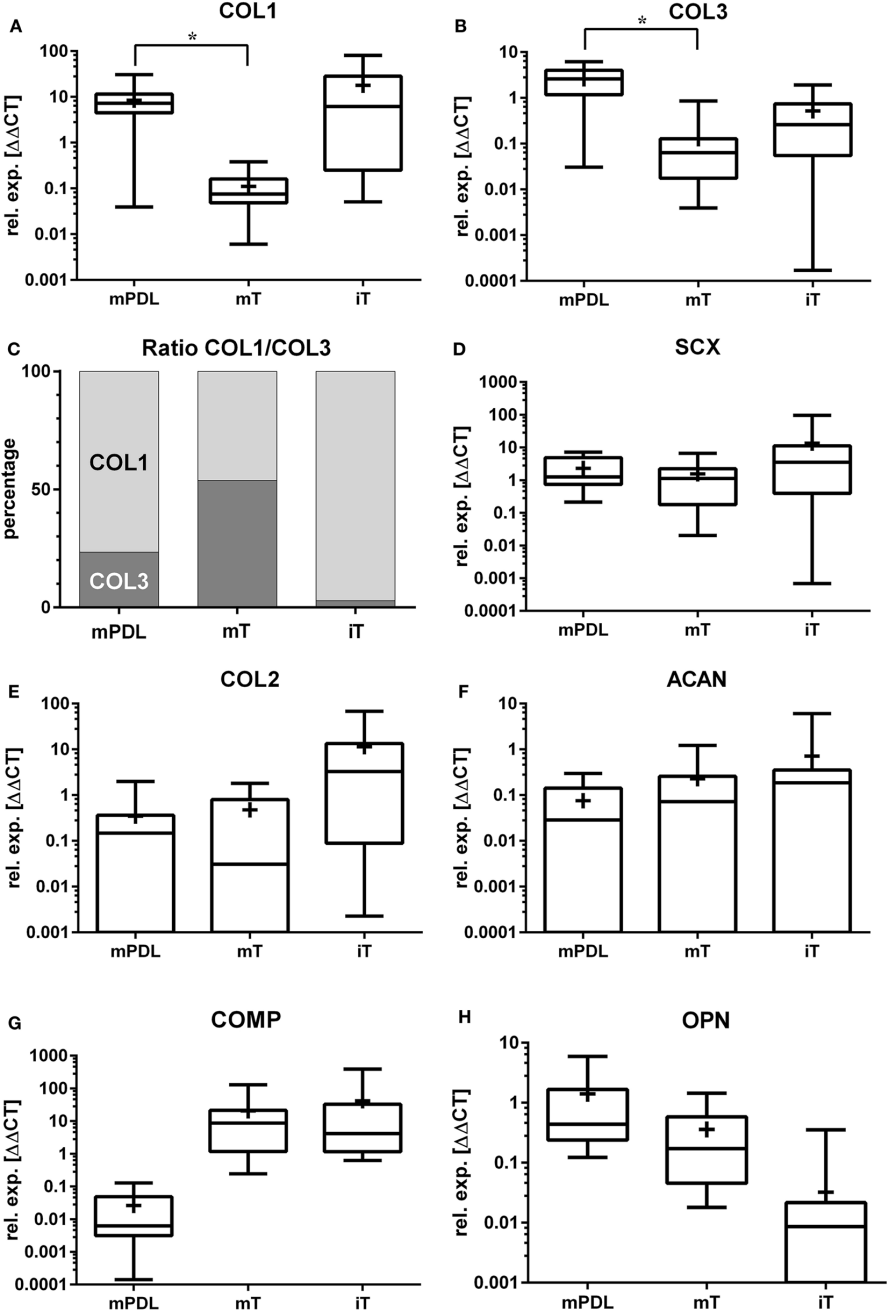
Relative gene expression (rel. exp. [ΔΔCt]) is presented as logarithmic application (log10) of the analyzed genes **(A)** *collagen type 1 alpha 1 (COL1)*, **(B)** *collagen type 3 alpha 1 (COL3)*, **(C)** ratio of mean relative expressions of *COL1/COL3*, **(D)** *scleraxis (SCX)*, **(E)** *collagen type 2 alpha 1 (COL2)*, **(F)** *aggrecan (ACAN)*, **(G)** *cartilage oligomeric matrix protein (COMP)*, and **(H)** *osteopontin (OPN)* in mature PDL (mPDL), mature tendon (mT), and immature tendon (iT). Mean relative gene expression is shown as plus (+). Statistical significance is shown as *p-value ≤0.05.

Significant differences were detected among the three differently located PDL samples from mature horses only for *COL2* gene expression (301, 306, 309). Among the five different tendon samples in mature horses, a significant difference was observed only for *ACAN* gene expression. Statistical analysis of *OPN* gene expression was not possible for adult tendons because of missing measurement values. Although there were significant differences within PDL and tendon samples, the mean gene expression values for all PDL and all tendon samples for each horse were calculated and the *t*-test for dependent samples was used to detect differences in gene expression between mature PDL and mature tendon (Figure 1).

### Two-Factorial ANOVA with Repeated Measurements

Except for *COL3*, no statistical differences were detected between mature and immature tendon samples taking differences between the locations into account. In terms of the global group differences, no statistical differences between mature and immature tendon could be detected. Analysis of *OPN* gene expression was not possible because of missing measurement values.

### Relative Gene Expression of *COL1*

The highest relative gene expression values for *COL1* were noted in immature tendon (Table 1; Figure 1A). The *COL1* expression in mature PDL was significantly higher than in mature tendon tissue (*p* < 0.05, Figure 1A).

**Table 1:** Mean, minimum, and maximum relative gene expression (ΔΔCt) of the analyzed genes [*collagen type 1 alpha 1 (COL1), collagen type 3 alpha 1 (COL3), scleraxis (SCX), aggrecan (ACAN), cartilage oligomeric matrix protein (COMP)*, and *osteopontin (OPN)]* in mature PDL (mPDL), mature tendon (mT), and immature tendon (iT).

		mPDL	mT	iT
COL1	Min–max	0.039–30.64	0.0061–0.38	0.051–80.57
	Mean	8.46	0.11	17.79

COL3	Min–max	0.031–6.19	0.0039–0.86	0.00017–1.91
	Mean	2.59	0.13	0.52

SCX	Min–max	0.21–7.19	0.02–6.59	0.00069–96.4
	Mean	2.29	1.55	13.5

COL2	Min–max	0–1.98	0–1.8	0.0023–67.5
	Mean	0.35	0.47	11.38

ACAN	Min–max	0–0.3	0–1.22	0–6.02
	Mean	0.075	0.23	0.71

COMP	Min–max	0.00014–0.13	0.25–128.8	0.62–388
	Mean	0.026	20.51	41.81

OPN	Min–max	0.12–5.91	0.018–1.45	0–0.35
	Mean	1.41	0.36	0.032

### Relative Gene Expression of *COL3*

The relative *COL3* expression in mature PDL was significantly higher than in mature tendon (*p* < 0.05, Figure 1B). Analysis of *COL3* expression levels revealed no statistical difference between mature tendon and immature tendon tissue (for global group differences).

### Ratio between Mean Relative *COL1* and *COL3* Gene Expressions

In the mature equine PDL, the ratio between the relative gene expression values for *COL1* and *COL3* was 3.3:1 (76.5–23.5%, Figure 1C). In the mature tendon, a *COL1* to *COL3* ratio of 0.9:1 (53.8–46.2%) was found, while in immature tendon the ratio was 34:1 (97.2–2.8%, Figure 1C).

### Relative Gene Expression of *SCX*

Gene expression of *SCX* exhibited no significant differences between mature PDL and mature tendon or between immature and mature tendon (Figure 1D). The expression values of *SCX* in immature tendon tissue were highly variable (Figure 1D; Table 1).

### Relative Gene Expression of *COL2*

The highest relative expression values for *COL2* were observed in immature tendon tissue (Figure 1E; Table 1). No statistical differences between gene expression in mature PDL and mature tendon or between immature and mature tendon were observed (Figure 1E).

### Relative Gene Expression of *ACAN*

The highest relative expression values, which were also highly variable, were noted in immature tendon tissue (Table 1). No statistical differences between *ACAN* gene expression in mature PDL and mature tendon or between immature and mature tendon were detected (Figure 1F).

### Relative Gene Expression of *COMP*

The highest relative expression values for *COMP*, which were also highly variable, were observed in immature tendon tissue (Table 1). No statistical differences between *COMP* gene expression in mature PDL and mature tendon or between immature and mature tendon were observed (Figure 1G).

### Relative Gene Expression of *OPN*

The highest relative gene expression values for *OPN* were seen in mature PDL (Table 1; Figure 1H). No statistical differences between *OPN* gene expression in mature PDL and mature tendon or between immature and mature tendon were detected (Figure 1H).

## Discussion

Currently, stem cell-based therapies for equine tendon regeneration are receiving increasing attention in the field of equine orthopedics (44, 45). Stem cells can be derived from different sources, i.e., bone marrow, adipose tissue, peripheral blood, etc. (46–51). Although promising results using those cells have been reported (52), it is still unclear how, and to what extent those cells contribute to tendon regeneration. In order to improve the efficiency of such cells, it has been suggested that stem cells could be induced to differentiate into the tenogenic lineage *in vitro*, prior to possible therapeutic use (47, 53). However, *in vitro* tenogenic pre-differentiation requires a high degree of technical effort. There are only a few reports of the practical use of such cells for regenerative therapies in horses (54, 55). Therefore, we aimed to find a source of cells that possess a wide range of regenerative properties and, at the same time, feature characteristics of tenogenic tissue. These prerequisites are broadly met by equine periodontal tissue cells. A promising study in a rat model gave the first positive indications for the use of PDL cells in tendon injuries (56). To confirm the suspected characteristics of such cells, functional mature equine PDL was compared to mature and immature tendon tissue at the molecular level.

In the equine PDL, continuous remodeling is required to accomplish permanent regeneration of the participating structures (37). For this reason, the PDL has a more cellular character than tendon tissue, and a high percentage of PDL cells are fibroblasts and fibrocytes (7). In the PDL of hypsodont mouse incisors, 50% of the volume is represented by fibroblast-like cells, while brachydont mouse molars showed a 10–15% smaller volume (35, 57). As a key factor for maintaining remodeling abilities in the PDL, a high population of fibroblasts is required (58). In brachydont molars of rats and mice, fibroblast proliferation rates of about 2% have been observed (39, 40). In the equine PDL, similar proliferation rates have been observed, indicating a relatively rapid turnover in comparison to tendon tissue, for example (37). Interestingly, in some regions of the equine PDL, proliferation rates of up to 4.5% have been measured (37). In contrast, tendon tissue has been described as a primarily static tissue (59, 60) with restricted regeneration capacity (14, 18, 19, 31–33, 59). About 90–95% of the tendon core collagen is not involved in turnover, and is in a stable state at adolescence, whereas only 5–10% of the collagen shows a higher rate of turnover (34). During maturation, cell proliferation is diminished and the number of stem cell progenitors decreases (59). Our observations in mature equine tendon showed that the expression of *COL1* and *3* is at a very basal level in this tissue. This result is consistent with the previously described characteristics of mature tendon tissue, indicating very limited collagen turnover. The relative gene expressions of *COL1* and *COL3* in mature tendon were similar (ratio 1.1:1), although, considering that COL1 is the main protein comprising the mature tendon, a considerably higher expression of *COL1* could be expected. This observation might be explained by the fact that collagen is accumulated in the developing tendon until a mature, more static state is reached (30, 61), and furthermore, limited turnover rates of collagens are detectable (34, 59). In the mature tendon, COL3 represents only a small amount of the ECM protein, whereas during tenogenesis, a considerably higher initial level of COL3 expression has been documented (62). During development, the COL3 expression decreases, while at the same time, the fibril diameters are increasing (62). These observations underline the function of COL3 as a crucial regulator during COL1 fibrillogenesis (62–64). Moreover, COL3 plays a key role during tendon healing and repair.

In contrast to the mature tendon, significantly higher expression levels of *COL1* and *3* characterize the mature equine PDL, indicating higher rates of collagen production and turnover. This finding is in line with previous studies that have documented a distinct spatial and temporal pattern of collagen remodeling in the equine PDL (37, 38). The relationship between expression of *COL1* and *COL3* in the mature PDL should be highlighted [ratio 3.3:1 (77–23%)]. The COL3 gene expression in the mature equine PDL is consistent with the COL3 protein content determined in the mature PDL of other hypsodont (65) and brachydont species (66). Similar COL3 expression has also been reported for fetal tissues (66–68). In this respect, the mature equine PDL seems to have embryonic-like properties and features high turnover rates.

In the immature tendon, the situation is quite different, with a striking expression of *COL1* and a comparatively lower expression of *COL3* (ratio 34:1). This result may reflect the fact that postnatal tendon growth predominantly occurs through an increase in collagen fiber diameter and length until the mature and more static state is reached (30, 61).

Another remarkable observation in our study was the fact that no statistical differences could be detected between the gene expression values for *SCX* in mature PDL and mature tendon tissue. *SCX* is a transcription factor that represents a precise marker of tendon and ligament progenitors as well as differentiated cells (22–24). SCX is, therefore, expressed in mature tendon tissue in which the inhibited tenocytes feature a highly bradytrophic metabolism, without any significant production of collagens in most areas (29, 30). Murchison and colleagues have described remarkable deficits in matrix and collagen fiber organization and a decreased number of tenocytes in SCX^−/−^ (knockout) mice flexor digitorum profundus tendons, which indicates the regulatory function of SCX in normal tendon development. A positive regulation of COL1 synthesis by *SCX* in tenocyte cells could also be demonstrated (24). Studies in mice molar PDL cells revealed similar gene expression levels for *SCX* in PDL cells compared to tenocytes (69). This observation is in agreement with our results. SCX was shown to regulate pro-α1 (I) collagen in tenocytes and to regulate COL1A1 transcription through binding to tendon specific element 2 (70). Maeda and colleagues demonstrated that a progressive loss in tensile load results in reduced *SCX* expression with a significant reduction in tendon COL1 fibrils (71). Moreover, the abovementioned study in rodent molar PDL cells revealed that orthodontic tooth movement, in other words, tensile forces on PDL cells, results in SCX expression (69). Takimoto and colleagues described an inhibitory effect of *SCX* overexpression on ECM mineralization and hypothesized possible functions of SCX as a regulator of PDL width and in prevention of ankyloses (69). Additionally, it was shown that SCX negatively regulates the expression of OPN, a mineral-associated protein, after osteogenic stimuli (69). OPN is a prominent marker for osteogenic differentiation. Our results lead to the suggestion that the initial definition of SCX as a marker for connective tissues that attach muscle to bone (22) might be extended to the PDL, as a tendon-like structure, which connects tooth to bone.

Osteogenesis-related genes can be detected at the tendon-bone insertion, and their expression is thus found in the calcified fibrocartilage zone of the entheses from mice (28). In our study, a remarkably high level of *OPN* expression was detected in the mature equine PDL. This observation might be a result of the sampling technique, if there was unintentional removal of osteoblasts from the side of the alveolar bone. Another possible explanation could be the suggestion made by Mori and colleagues that OPN functions in the regulation of tissue remodeling (72).

Local compression loads can induce tendon tissue to produce cartilage-like matrix (26) and subsequently transform it into fibrous cartilage tissue, e.g., in tendons that wrap around bony or fibrous pulleys (15). More precisely, pressurized regions within tendons can undergo a local transformation from tight collagenous tendon tissue into fibrocartilage. This conversion can also be described as chondrogenic metaplasia. Therefore, we examined the expression of characteristic fibrocartilage genes like *COL2, COMP*, and *ACAN*. Fibrocartilage can be found within fascicles, and in the endo- or epitenon, where it helps to prevent blood vessels from compression stresses or contributes to improved sliding between fascicles (15). Furthermore, it is supposed that tendon tissue is generally able to synthesize a cartilage-like matrix along the tendon to adapt to changing mechanical conditions (26). Considering the position of the equine PDL (between the teeth and the alveolar bone) and its biomechanical environment, in which remarkable tensile and compressive stresses occur (10, 73, 74), chondrogenic metaplasia should be expected. However, it has not been documented in the literature nor was gene expression related to chondrogenic metaplasia detected in this study. There has been speculation that the PDL possesses distinct mechanisms that prevent functionally disadvantageous chondrogenic metaplasia. A possible explanation is provided by the current opinion that epithelial cell rests of Malassez perform an important function in maintaining PDL tissue homeostasis (75). It is believed that epithelial cell rests of Malassez counteract ankylosis by maintaining the PDL space (75). The ECM in fibrocartilaginous regions often comprises ACAN and COL2. These components enable tendons to absorb water and resist compression stresses (15). COL2 distribution varies between different tendons and subjects, presumably dependent on differing load and compression forces (15). In the present study, although no differences in *ACAN* or *COL2* gene expression were detected between the equine PDL, and the mature and immature tendon, the high variability in the expression levels between immature specimens are noteworthy. However, a functional explanation remains open.

In summary, scientific interest in understanding molecular-, growth-, and turnover mechanisms in tendons remains high, with the aim of obtaining important information about healing processes and new therapies. Tendon injuries represent a far-ranging problem in equine orthopedics. The observed low collagen expression rates in mature tendon tissue indicate limited turnover. This circumstance, together with the minor cellularity and vascularity, leads to poor regeneration capacity in tendon tissue. In contrast, the mature equine PDL can be described as a highly dynamic system, with high collagen expression contributing to continuous remodeling. On the molecular level, the equine PDL exhibits a tendon related gene expression profile. Based on our preliminary results, additional studies are required to further characterize equine PDL-derived MSC and to evaluate their possible use in cell-based regenerative therapies.

## Ethics Statement

According to German legislation, the postmortem harvest of specimens does not need any permission of the animal welfare authority. The former owners of the horses consent to post mortem dissection.

## Author Contributions

AP and CS designed the study. AP, CS and BK collected the specimens. AP and BK conducted the molecular biological analysis. AP, KF and BK performed the statistical analysis. AP assembled the results. AP prepared and edited the manuscript. CS and BK edited the manuscript. All authors read and approved the final version of the manuscript.

## Conflict of Interest Statement

None of the authors of this paper has a financial or personal relationship with other people or organizations that could inappropriately influence or bias the content of the paper.
